# Life-history of *Palaeoloxodon antiquus* reveals Middle Pleistocene glacial refugium in the Megalopolis basin, Greece

**DOI:** 10.1038/s41598-024-51592-9

**Published:** 2024-01-16

**Authors:** Effrosyni Roditi, Hervé Bocherens, George E. Konidaris, Athanassios Athanassiou, Vangelis Tourloukis, Panagiotis Karkanas, Eleni Panagopoulou, Katerina Harvati

**Affiliations:** 1 Paleoanthropology, Institute for Archaeological Sciences, Department of Geosciences, Eberhard Karls University of Tübingen, Tübingen, Germany; 2 Biogeology, Department of Geosciences, Eberhard Karls University of Tübingen, Tübingen, Germany; 3 Senckenberg Centre for Human Evolution and Paleoenvironment, University of Tübingen, Tübingen, Germany; 4 Hellenic Ministry of Culture, Ephorate of Paleoanthropology–Speleology, Athens, Greece; 5 Department of History and Archaeology, School of Philosophy, University of Ioannina, Ioannina, Greece; 6 DFG Centre for Advanced Studies ‘Words, Bones, Genes, Tools’, Eberhard Karls University of Tübingen, Tübingen, Germany; 7 M.H. Wiener Laboratory for Archaeological Science, American School of Classical Studies, Athens, Greece

**Keywords:** Palaeoclimate, Biochemistry, Biogeochemistry, Ecology, Palaeoecology, Stable isotope analysis, Palaeontology

## Abstract

The Balkans are considered a major glacial refugium where flora and fauna survived glacial periods and repopulated the rest of Europe during interglacials. While it is also thought to have harboured Pleistocene human populations, evidence linking human activity, paleoenvironmental indicators and a secure temporal placement to glacial periods is scant. Here, we present the first intra-tooth multi-isotope analysis for the European straight-tusked elephant *Palaeoloxodon antiquus*, on an adult male individual excavated in association with lithic artefacts at the MIS 12 site Marathousa 1 (Megalopolis basin, Greece). The studied find also exhibits anthropogenic modifications, providing direct evidence of hominin presence. We employed strontium, carbon and oxygen isotope analysis on enamel bioapatite to investigate its foraging and mobility behaviour, using a sequential sampling strategy along the tooth growth axis of the third upper molar, to assess ecological changes during the last decade of life. We found a geographically restricted range, in a C_3_-dominated open woodland environment, and relatively stable conditions over the examined timeframe. Our results show that, despite the severity of the MIS 12 glacial, the Megalopolis basin sustained a mesic habitat, sufficient plant cover and limited seasonal fluctuations in resource availability, pointing to its role as a glacial refugium for both fauna and hominins.

The Balkan peninsula is considered to have played a major biogeographic role in Pleistocene Europe, broadly acting as a glacial refugium for both plant and animal species, as well as a source for the repopulation of higher latitude regions during interglacial periods (e.g.,^1–6^). In recent years, the Megalopolis basin, southern Greece, has emerged as a potential refugium-within-a-refugium area^7^ (or micro-refugium^6^), harbouring favourable environmental conditions, freshwater resources, rich faunal and floral communities, as well as human populations in the Middle Pleistocene^8–15^. Here, we investigate the life history of a straight-tusked elephant (*Palaeoloxodon antiquus*) from Marathousa 1 (hereafter MAR-1; Fig. 1; Supplementary Notes 2), a Lower Paleolithic elephant butchering site dating to the MIS 12 (∼478–424 ka BP) glacial^8,11,12,16–18^, one of the most impactful glacial intervals, which featured the largest ice volume throughout the Quaternary^19–21^. The MIS 12 cold and arid conditions resulted in vegetational shifts, such as the retraction of temperate forests and expansion of steppe vegetation^21–24^ and marked a turning point for hominin adaptations reflected in their subsequent (MIS 11) demographic expansion and the dissemination of diverse locally-developed technological innovations (e.g. refs.^25–29^). In addition to rich cultural remains, comprising more than 2000 stone and bone artifacts^13^, MAR-1 has yielded a diverse faunal assemblage, including an elephant partial skeleton (hereafter MAR-1A-5). The MAR-1A-5 preserves anthropogenic cutmarks and was found associated with lithic artefacts, thus providing a direct link to hominin activity at the site^10,13^. We combine carbon (^13^C/^12^C), oxygen (^18^O/^16^O), and strontium (^87^Sr/^86^Sr) isotope analyses to investigate the foraging and mobility patterns of this individual, and to help reconstruct paleoenvironmental conditions in the basin during its lifetime.

**Figure 1:**
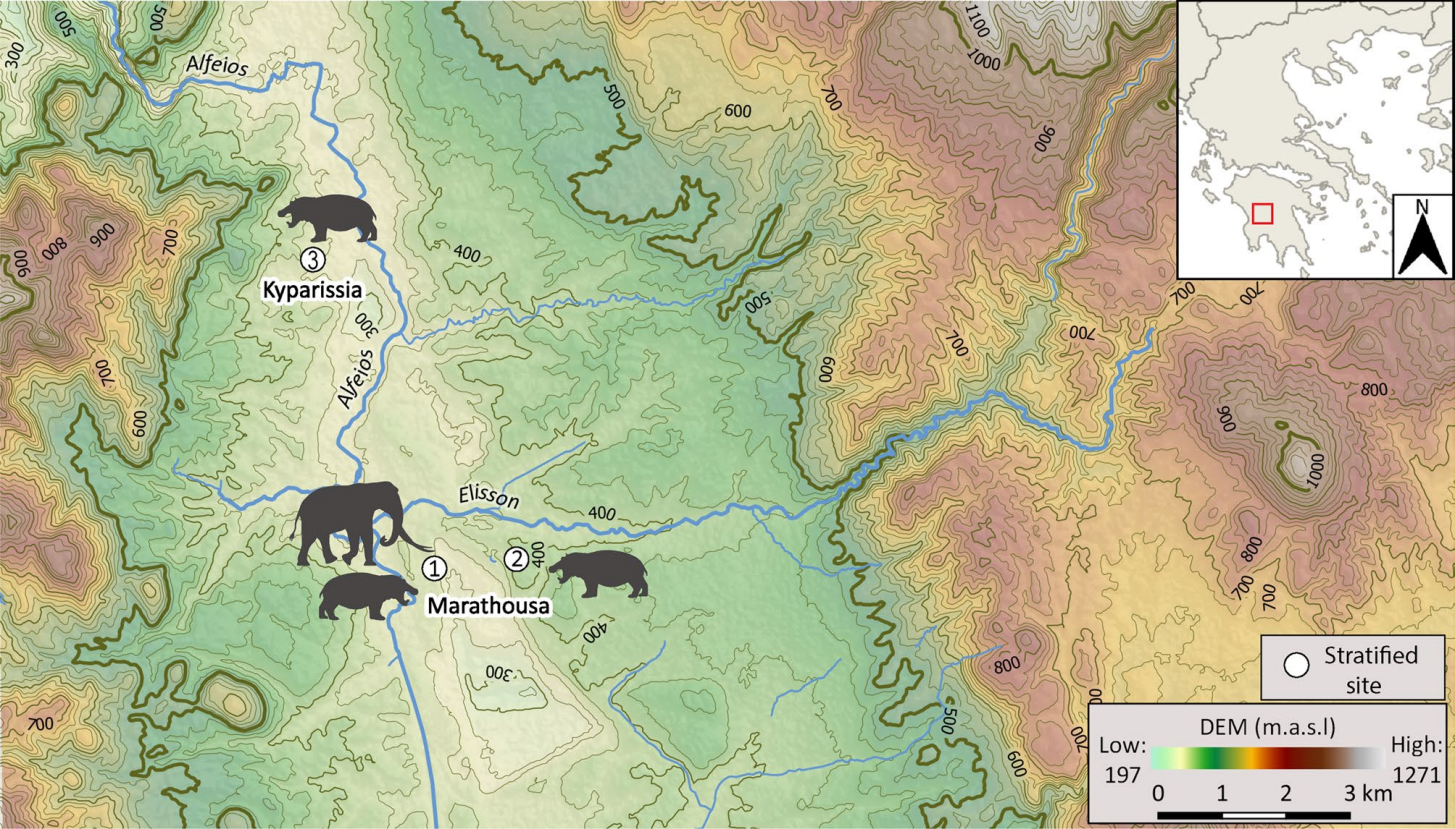
Topographic map of the Megalopolis basin showing the location of the sites: (1) Marathousa 1; (2) Marathousa 2; (3) Kyparissia 4. Contour interval: 25 m. Digital Elevation Model (DEM) from the Shuttle Radar Topography Mission (SRTM) version 3^126^. The map was created using QGIS version 3.16 (https://www.qgis.org).

Straight-tusked elephants (genus *Palaeoloxodon*) originated in Africa and dispersed to Eurasia towards the end of the Early Pleistocene^30^, becoming common in Eurasian Middle–Late Pleistocene assemblages. The European species, *P. antiquus,* occupied a broad spectrum of habitats^31,32^, demonstrating high dietary flexibility and foraging behaviour, ranging from exclusive browsing to grazing, depending on the available resources^33,34^. Despite its ecological adaptability, it is generally associated with temperate climatic conditions^32^. In central Europe, it was widely distributed during interglacial or interstadial phases, while during glacial stages, its range was mostly restricted to southern Europe^35–38^. Its occurrence is widely documented in Greece, including the Megalopolis basin^37,39^. In MAR-1 at least two individuals have been discovered during excavation, both preserving evidence of anthropogenic modifications^10^.

Proboscideans played an important role in the adaptations and subsistence of Paleolithic hominins^10,40–42^. The geographic distribution and ecological signal of *P. antiquus* largely overlapped with the ecological preferences of Middle Pleistocene hominins^42^. Due to its high fat and meat quantity it likely constituted a crucial component in their diet^43,44^. Remains of *P. antiquus* associated with hominin activity either directly (e.g., cut marks, bone breakages, artefacts made from proboscidean bone) or indirectly (contextual linkage of carcasses with cultural finds), are common in the Middle Pleistocene record of western Eurasia^42^. Such associations allow paleoecological investigations of proboscidean diets and habitats to shed light also on hominin environments and subsistence (e.g.,^45,46^). Here we show that the MIS 12 individual MAR-1A-5, and, by extension, the hominins that processed its carcass, lived in relatively stable conditions with sufficient plant cover and limited seasonality.

## Results

We sampled sequentially the second distalmost lamella of the MAR-1A-5 upper right M3, representing approximately the last ten years of the individual’s lifespan, to obtain intra-tooth strontium, carbon and oxygen isotopic profiles. This multi-proxy strategy targeted the reconstruction of MAR-1A-5’s mobility, diet and habitat, and paleoclimate, respectively. For the strontium analysis, the baseline relied on published data covering the Peloponnese^47^, as well as on fossil *Hippopotamus* samples from three Middle Pleistocene localities of the Megalopolis basin. No significant correlation was observed between Sr–C or between Sr–O isotopes in the sequential profile.

### Strontium isotopes

**Figure 2:**
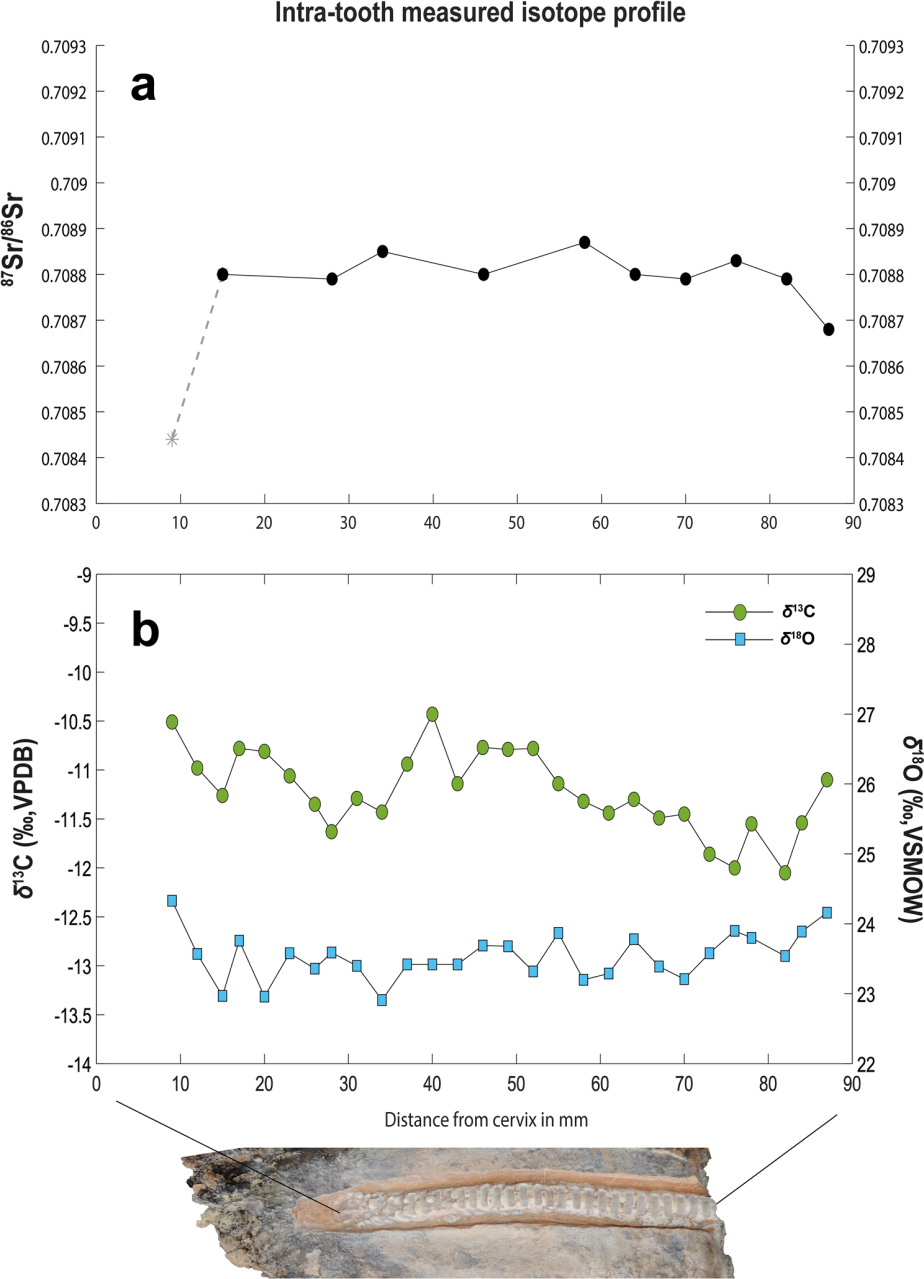
Enamel intra-tooth strontium isotope ratios (**a**) and intra-tooth carbon and oxygen stable isotope results (**b**) for the *Palaeoloxodon antiquus* of Marathousa 1. In (**a**), the gray dashed line represents an outlier.

^87^Sr/^86^Sr ratios were relatively homogenous for the majority of the profile (0.70868–0.70887; variability of 0.00019), with the exception of sample 28, taken closest to the cervix (Fig. 2A & SU Table 1; 0.70844 ± 0.00003). This result was likely related to differences in enamel maturation and/or diagenesis. We therefore considered it as an outlier and excluded it from further analysis. The remaining ratios approximated the value obtained from the fossil *Hippopotamus antiquus* excavated at the same site (MAR-1B-8; 0.70870 ± 0.00002). The ^87^Sr/^86^Sr ratio from the *H. antiquus* specimen MAR-2B-2 from the broadly contemporaneous nearby locality Marathousa 2^14^ was notably higher (0.70906 ± 0.00003), as expected for an individual feeding near the outcrops of the Tripolis geotectonic unit^47^; whereas the value obtained from the Kyparissia 4 *H. antiquus*^48^ (KYP4A-1004) (0.70838 ± 0.00001) was consistent with the strontium range of the Pindos geotectonic unit^47^. These results were in agreement with the localities’ respective geographic locations (See Supplementary Fig. 1).

### Measured carbon and oxygen isotopic values

Measured *δ*^13^C values ranged between –10.4 and –12.1‰ VPDB (mean –11.2‰ VPDB) (Full data in Supplementary Table 1). The amplitude of variation (1.62‰) indicated relatively low intra-tooth variability. Nevertheless, a quasi-sinusoidal trend was observed both in the intra-tooth profile and in the moving average of *δ*^13^C values. This trend was expressed by at least two downward and one upward periods occurring on an inter-annual scale (Fig. 2B).

Measured *δ*^18^Ο values ranged between + 22.9‰ and + 24.3‰ VSMOW (mean + 23.5‰ VSMOW) (Supplementary Table 1). A sinusoidal pattern was also observed in the oxygen isotope time series, with fluctuations occurring at relatively regular intervals (Fig. 2B). However, their low amplitude of variation (1.42‰), suggested an attenuated seasonal signal or, alternatively, a lack of seasonal effects in the isotopic composition of the local meteoric water and, subsequently, in the animal’s body fluids for the measured enamel *δ*^18^O series.

### Modelled carbon and oxygen isotopic values

**Figure 3:**
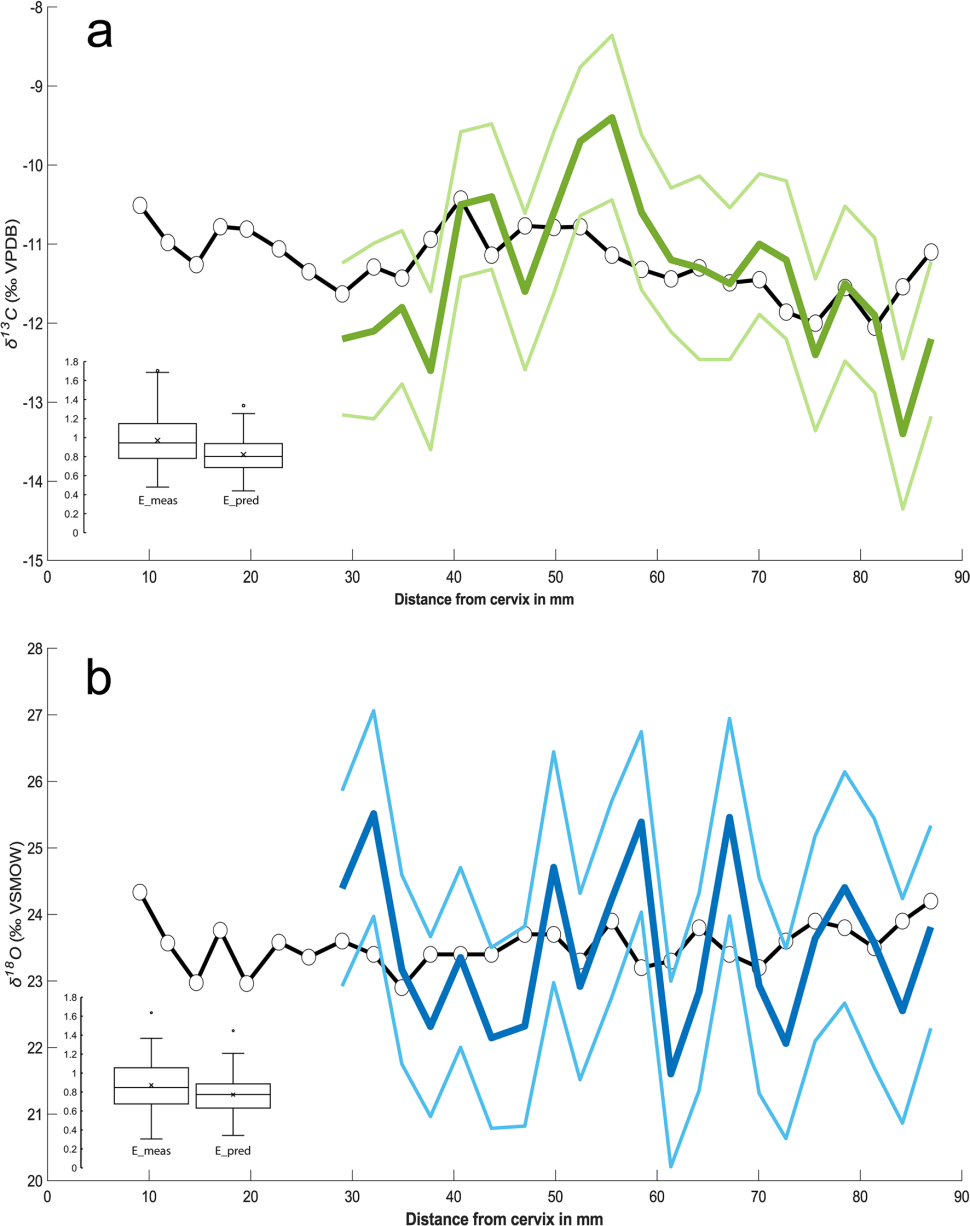
Inverse model results for *δ*^13^C (**a**) and *δ*^18^Ο (**b**). Bold coloured lines represent the average estimated input signal from 100 solutions (± 1σ) and circles represent measured stable isotope data. In both models l_a_ = 30 mm, l_m_ = 70 mm, openindx = 1, r2(σ_Δx_) = 0.3 mm, and r3(σ_Δz_) = 0.5 mm. For (A), r1 = 0.1‰, ε^2^ = 0.007, and the reference vector (RV) =  − 12.1 to − 10.4‰. For (B) r1 = 0.2‰, ε^2^ = 0.003, and RV =  + 22.9 to + 24.2‰. Data from within 25 mm of the cervix are excluded from the inversion. Boxplots show the comparison between measured error (E_meas) and predicted error (E_pred) (see ref.^49^). The climaxes identified in the *δ*^18^Ο series, which coincide with climaxes in *δ*^13^C values, likely reflect summer conditions, whereas estimated minima in *δ*^18^Ο and the corresponding *δ*^13^C troughs express winter-time conditions over a period of ~ 5 years (from 28 to 87 mm along the profile).

Sampling geometry and differential enamel formation processes can dampen the amplitude of the primary dietary and body water signal. We employed inverse modelling to reduce such effects^49,50^. This increased the range of carbon intra-tooth variability to 4‰. The magnitude of variation between two successive wave heights ranged from 1.4‰ to 2.1‰, suggesting moderate intensity of sub-annual isotopic change. The moving average of the modelled carbon profile (28–87 mm from the cervix) showed 5 peaks and 5 troughs. Additionally, a trend towards more positive *δ*^13^C values was observed in the earlier forming section of the lamella (from the apex until approximately 56 mm in the profile), with carbon values progressively declining thereafter (Fig. 3A). Hence, the multi-seasonal pattern was prominent in both measured and modelled carbon enamel data. All carbon values fell within the expected range for a diet consisting of C_3_ vegetation (see Methods).

The modelled *δ*^18^Ο data showed a larger range of values (+ 21.6‰ – + 25.5‰) and more pronounced fluctuations, which appeared relatively regularly at a distance from 28 to 87 mm from the cervix (Fig. 3B). The estimated amplitude in adjacent wave heights ranged between 1.9 and 3.9‰.

## Discussion

**Figure 4:**
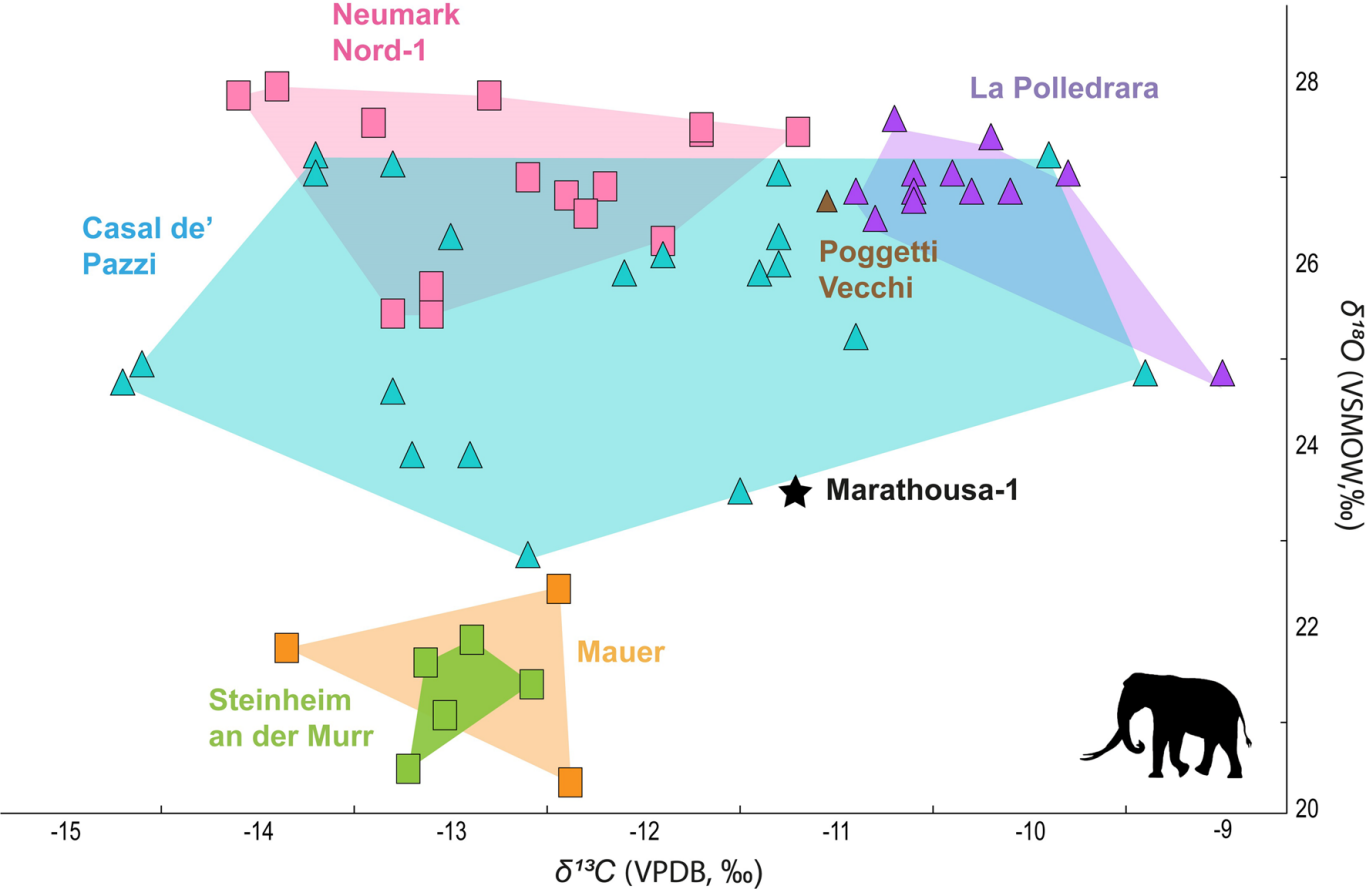
Bivariate plot showing *δ*^13^C_sc_ (VPDB) and *δ*^18^Ο_sc_ (VSMOW) values for *Palaeoloxodon antiquus* in European Middle Pleistocene localities. For details of the studied localities and the data used for the inter-site comparison see Methods and Supplementary Table 2. Convex hulls are used as visual representations of the range of isotopic values between different sites and have no statistical meaning.

The *P. antiquus* foraging ecology remains relatively poorly understood. Most studies as yet have focused on dental micro- and mesowear, thus providing information for a specific temporal frame that captures the diet of the last days/weeks before the death of the individual or the average annual diet, respectively^33,34,51–55^. Stable isotope analyses for the species, which offer more long-term foraging inferences, are scarce. Published isotopic data from six Pleistocene European localities provided a comparative framework for our analysis (see Online methods and SU Table 2). Compared to *P. antiquus* from Italy, the MAR-1A-5 average *δ*^13^C value approximates carbon values from MIS 7 interglacial individuals^53,56,57^, indicating a similar mesic/open woodland habitat with glacial MIS 12 Megalopolis basin. In contrast, the MAR-1A-5 carbon values are lower than those recorded from MIS 9 interglacial individuals^53^, suggesting a more densely forested, humid habitat in Megalopolis. Furthermore, when compared to MIS 5, 11 and 15 interglacial specimens from Germany^54,58^, the MAR-1A-5 carbon values point to more open / drier conditions. In terms of climate, the MAR-1A-5 mean *δ*^18^Ο value indicates a cooler and / or more humid setting than either MIS 7 or MIS 9 at the Italian sites^53,56,57^, but warmer and / or drier conditions than two Middle Pleistocene interglacial German localities^58^ (Fig. 4; see also SU 4). The inter-site comparison of the currently available *P. antiquus* stable isotope data agrees with the correlation of the MAR-1 archaeological sequence with a glacial stage (MIS 12), reflecting colder conditions in the eastern European peri-Mediterranean region. Nevertheless, the MAR-1A-5 isotopic composition also indicates the persistence of a moderately humid setting in the Megalopolis basin, supporting sufficient amounts of C_3_ vegetation cover.

Carbon isotope variability can be used to infer vegetation shifts and possible seasonality. Our measured carbon isotope intra-tooth series indicates periodic variability, which is stronger on a multi-annual rather than a sub-annual temporal scale. The trend persists also in the modelled carbon isotopic composition albeit with an increase from a low to a moderate magnitude of variation. These fluctuations can be related to environmental or dietary patterns, as inherent seasonal fluctuations in the isotopic composition of C_3_ plants, but also variation between plant parts (i.e., leaves relative to seeds and flowers) can result in differences of 1–3‰ in plant *δ*^13^C^59^. The temporal interval of change in the isotopic profile of MAR-1A-5 suggests that, although low to moderate seasonal effects may have conditioned the isotopic composition of its diet and body water, multi-season habitat or dietary shifts were equally, if not more, pronounced. Climate variability, for example prolonged changes in water availability and precipitation lasting several years, is unlikely to underlie the carbon isotope trend, as such shifts would also manifest in the *δ*^18^O series but are lacking in our data. The long-term fluctuations in the *δ*^13^C series may therefore be ascribed to multi-annual ranging mobility between different microhabitats or plant communities with varying canopy cover within the basin, while still using local water-sources with similar oxygen isotopic composition. In this scenario, the consistency of ^87^Sr/^86^Sr ratios may reflect the geological homogeneity of the area or, alternatively, the slow enamel maturation processes, resulting in strontium averaging and thus hindering the identification of short-scale nomadic events.

The measured enamel *δ*^18^Ο profile yielded an attenuated signal, which could be interpreted as low seasonality in precipitation and temperature. However, such a pattern may also be produced by dampening related to hydrological processes (i.e., drinking from long-lived resident water bodies, such as lakes^60^). A recent multi-proxy study on the water-level history of MAR-1 showed that, at the time of deposition of the MAR-1A-5 elephant skeleton, seasonal ponds had developed under a mostly dry and cold climate, conditions which persisted over ∼10 ka^8^. We therefore consider signal dampening due to such hydrological processes unlikely. Rather, the observed low amplitude of variation is in part influenced by the geometry of sampling and differential enamel formation processes along the tooth growth axis^50,61,62^. When inverse modelling was undertaken to minimise these effects, stronger fluctuations in the oxygen isotope time series were revealed (~ 4‰). Nevertheless, even the modelled oxygen data are inconsistent with strong seasonal fluctuations in rainfall and temperature in the MIS 12 Megalopolis basin. Even taking the difference between modelled and measured enamel data into account, the amplitude of seasonal oxygen variation for MAR-1A-5 is still much lower compared to seasonal data obtained from North American mammoths and mastodons (intra-tooth variation of 6–11‰)^63–65^, further supporting moderate seasonality for MIS 12 Marathousa 1.

Strontium isotopes are used to infer animal mobility patterns. Both the MAR-1A-5 strontium ratios and those of the three Middle Pleistocene *Hippopotamus* specimens analysed fall within the range of published environmental strontium values of the Tripolis and Pindos geotectonic units^47^, the local predominant geological outcrops. The relative consistency of the MAR-1A-5 strontium ratios indicates that the Marathousa elephant likely maintained strong site fidelity (i.e., a tendency to occupy the same area over a larger time frame^66^) during the decade represented by our samples, staying within the Megalopolis catchment.

In summary, our multi-isotope incremental approach indicates that MAR-1A-5 maintained a limited home range and did not exhibit large-distance mobility. It likely responded to differential seasonal food availability by consuming relatively more grasses during the wet season and relatively more woody, fruity, or leafy vegetation during drier months. Similar behavioural patterns are observed in African savannah and forest elephants (*Loxodonta africana, Loxodonta cyclotis*)^67–70^ and Asian elephants (*Elephas maximus*)^71,72^. The isotopic data also show that seasonal effects, both in climatic variables and in plant productivity, were moderate, therefore ensuring essential forage within the Megalopolis catchment throughout the year over the examined time frame. Therefore, a scenario of nomadic mobility, *i.e.,* irregular inter- or intra-annual movement patterns within the Megalopolis basin, is most likely for MAR-1A-5. Such a hypothesised pattern of long-term recursion —the return to a previously utilised foraging patch after a long time— has been observed in Asian^73^ and African elephants^74^. This behavior is attributed to vegetation regeneration and resource replenishing of previously overutilized patches, highlighting the strong influence of spatial memory of available resources in the movement decisions of elephants^73–78^. Although extant proboscideans have also been reported to travel over long distances (> 500 km), such long ranging behaviour is associated with severe environmental stress and habitat degradation^79,80^. The lack of long-distance ranging in our data, therefore, further points to environmental stability over the time period sampled.

The locality MAR-1 is correlated to MIS 12, considered the coldest Pleistocene glacial episode and coinciding with the largest expansion of ice cover in northern and eastern Europe. In Greece, it resulted in the formation of large ice fields on Mount Tymphi and the northern slopes of Mount Smolikas, as well as on Mount Chelmos in the Peloponnese^81,82^. Pollen records from Tenaghi Philippon (north-east Greece)^24^ and Lake Ohrid (North Macedonia-Albania)^21^ show that arboreal pollen reached its absolute minimum during the MIS 12 glacial maximum. This indicates extreme contraction of tree populations even in southern areas of the Balkans, considered a principal glacial refugium of Europe. Nevertheless, the prevalent climatic and environmental conditions of the Megalopolis basin remained suitable for the habitation of *P. antiquus*, a species commonly associated with temperate climates, forests, and warmer conditions. The continuous presence of *P. antiquus* through multiple periods in the Megalopolis basin (^10,83–85^, ongoing studies) highlights the resilience and adaptability of the species, which survived under diverse climatic conditions and occupied a broad range of habitats, including woodland and open grassland.

Multiple proxies have been employed to reconstruct the paleoenvironmental conditions of the archaeological layers at MAR-1. Phytoliths, diatoms and plant macrofossils demonstrated that the surrounding vegetation was diverse, suggesting still water conditions and a depositional context near a marginal reed swamp. The occurrence of plant taxa such as Palmae, *Acer, Quercus, Ulmus, Salvinia natans,* stratigraphically associated with MAR-1A-5, indicates a temperate climate at the time of the elephant’s death, with mixed habitat components of dry and damp woodland, reed swamp, open damp ground, and open water^9^. The vertebrates recorded at the site comprise terrestrial (e.g., *Macaca, Bison, Cervus, Dama, Canis*) and semi-aquatic mammals (e.g., *Castor, Hippopotamus, Lutra*)^10,15^ and freshwater birds (e.g., *Anas)*^86^*.* It is indicative of a landscape with substantial woodland components as well as more open areas and the existence of a permanent freshwater body with mean summer temperatures reaching 10–15 °C (approximately 11 °C colder than present-day summer temperatures) in the Megalopolis basin^8,10^. These paleoenvironmental interpretations are consistent with a C_3_-dominated ecosystem consisting of C_3_ woodland/open grassland under mesic conditions and low to moderate seasonality indicated by our isotopic analysis.

The effects of the MIS 12 glaciation in the Megalopolis area therefore appear relatively mild, maintaining conditions that allowed the persistence of a diverse temperate fauna and flora, including elephants, monkeys, hippopotami and deer, some of them exploited by Middle Pleistocene hominins^10,14^. Additionally, habitat heterogeneity and moderate seasonality, as indicated by the composition of the floral and faunal indices and the present isotope analysis, provided diverse foraging options to mammalian species, including hominins, facilitating the persistence of organisms with different ecological and dietary requirements.

## Conclusions

Our study of the MAR-1A-5 *Palaeoloxodon antiquus* from the MAR-1 Lower Paleolithic elephant butchering site (Megalopolis basin, southern Greece) provides the first intra-tooth multi-isotope analysis of the European straight-tusked elephant. It is also the first stable isotope study of this species from a peri-Mediterranean glacial context, shedding new light on the foraging ecology and habitat of both this emblematic megaherbivore and the hominins exploiting it. The decadal and sub-annual scale inferences provided by our detailed life history reconstruction of MAR-1A-5 add new high-resolution detail to the paleoenvironmental reconstruction of the Megalopolis basin during MIS 12, one of the most impactful glacial periods in Europe. Our results strongly support the hypothesised role of the Megalopolis basin as a ‘refugium-within-a-refugium’^7^, a distinct refugial area within the major Balkan glacial refugium, where not only a diverse temperate fauna and flora, but also hominins, persisted even during the harshest glacial periods of the Middle Pleistocene.

## Methods

### Sampling and proboscidean enamel formation

Enamel samples were obtained from the second distalmost lamella of the fragmented upper right third molar (M3). The molars of adult elephants are formed over a timespan of approximately 10–12 years. Plate formation is sequential and proceeds on a mesial–distal direction^87^, wherein the resulting effect is the development of more pronounced dental wear in the mesial part of the tooth compared to the distal one. Hence, sequential sampling of the distal lamellae is intended to provide a longer isotopic record.

Stable isotope ratios for chemical elements incorporated in tooth enamel matrix during enamel formation do not remodel once mineralization is completed. Therefore, the isotopic composition of incremental samples in hypsodont teeth, such as elephantid molars, is considered to record annual or sub-annual climatic and dietary shifts, whose temporal resolution depends on the species-specific enamel secretion and mineralization times, as well as on the implemented sampling design^88–90^. The vertical extension rate of elephantid molar plates, i.e., in the occlusal–basal direction, has been estimated to approximately 15–22 mm/year^61,91–93^. At the same time, amelogenesis, the process with which enamel forms, occurs at two stages. The secretory stage involves the incremental deposition of enamel matrix by ameloblasts, which transpires at a low angle from the enamel-dentine junction (EDJ) to the outermost surface^61^. The second stage involves the prolonged maturation of enamel, during which enamel crystals become progressively denser. This latter process results in signal attenuation and lower temporal resolution pertaining to both the timing of enamel maturation, as well as sampling geometry^88,94^, although the extent of dampening is lately considered smaller than originally predicted^95^.

Here, we followed an incremental sampling strategy to generate intra-tooth isotopic profiles, wherein several samples were obtained along the tooth growth axis in 2–3 mm intervals and ~ 20 mg of powdered enamel was extracted for each sample across the full enamel thickness. Prior to sampling, a section of tooth cementum over the area of interest was mechanically removed using a handheld rotary tool equipped with a diamond burr to expose the enamel. In addition, approximately 1 mm of the outer enamel surface was drilled away, to remove impurities and minimize the risk of sample contamination.

## Inverse modelling

In order to minimize signal attenuation and obtain a more reliable representation of the primary isotopic input signal through the application of conventional sampling, we applied the inverse modelling procedure developed by Passey et al.^49^ using the code provided by the authors. The model accounts for the contribution of isotopic signals from adjacent enamel increments that formed during different seasons and, through mathematical inversion, produces an estimated input signal that better reflects the amplitude of variation in the isotopic composition of diet and drinking water. Model input parameters include measured isotope ratios, sample geometry (length and depth), and their respective measurement uncertainties (1σ), as well as parameters related to enamel formation, namely the length of apposition (l_a_), length of maturation (l_m_) and initial mineral content during enamel deposition (f_i_). To measure sampling geometry with maximized accuracy, a silicon mould of the entire sampled profile was sectioned and scanned with a flatbed-scanner. Measurements were obtained using the software ImageJ^96^. With respect to enamel formation parameters, we used values established for the extant African savannah elephant *Loxodonta africana* and the extinct Columbian mammoth *Mammuthus columbi* as proxies for *P. antiquus*, where l_m_ = 70 mm, f_i_ = 65% FD, and l_a_ = 30 mm^50^. Samples obtained within 25 mm from the cervix were excluded from the modelling procedure, as suggested by Uno et al.^50^, due to changes in enamel growth rate and geometry close to the root apex^50,93^. Conventional sampling at intervals of 2–3 mm on molars of the Elephantinae subfamily followed by the application of inverse modelling is considered sufficient for the recovery of sub-annual trends^50^.

## Chemical pretreatment and analysis

The acquired samples were chemically treated at the Biogeology laboratory of the University of Tübingen (Germany). Following the protocol described by Bocherens et al.^97^; Koch et al.^98^, the sampled enamel powder was reacted with 2.5% sodium hypochlorite (NaOCl) for 24 h to remove organic components. After the reaction time, the vial contents were centrifuged at 3.500 rounds/minute for 3 min, the supernatant was removed, and the contents were rinsed repeatedly with distilled water. Next, samples were soaked to 1 M acetic acid buffer and left to react for 24 h, to remove non-structural, diagenetic carbonates. Once the supernatant was removed, the powder was rinsed repeatedly with Milli-Q H_2_O and dried at 35° C for 72 h. Around 3 mg of purified biogenic apatite obtained through this process was reacted with concentrated (99%) orthophosphoric acid (H_3_PO_4_) for 4 h at 70 °C. Carbon and oxygen stable isotope ratios were obtained by analysing the gaseous CO_2_ reaction products using a Multi-Flow-Geo interfaced with the Elementar Iso Prime 100 IRMS. Two international standards, IAEA-603 (International Atomic Energy Agency; *δ*^13^C =  + 2.46‰, *δ*^18^O = –2.37‰, relative to VPDB) and NBS-18 (National Bureau of Standards, now National Institute of Standards and Technology or NIST; *δ*^13^C = –5.00‰ and *δ*^18^O = –22.96‰, relative to VPDB), as well as three in-house standards (Elephant CRM, Hippo CRM, Laaser Marmor CRM), were used for calibration. The analytical precision of the measurements is higher than 0.1‰ and 0.2‰ for carbon and oxygen respectively, based on multiple isotopic analysis of modern tooth enamel of elephant and hippopotamus prepared and analysed at the same time as the fossil samples. Carbon and oxygen stable isotope results are expressed using the standard *δ* notation:

$$\delta ^{{\text{j}}} {\text{X}} = \frac{{\left( {^{{\text{j}}} {\text{X}}/^{{\text{i}}} {\text{X}}} \right)_{{{\text{sample}}}} }}{{\left( {^{{\text{j}}} {\text{X}}/^{{\text{i}}} {\text{X}}} \right)_{{{\text{standard}}}} }} - 1$$

where jX is the heavier isotope and iX is the lighter isotope^99^. Measured ratios refer to ^13^C/^12^C VPDB or ^18^O/^16^O VSMOW, wherein VPDB is Vienna Peedee Belemnite and VSMOW is Vienna Standard Mean Ocean Water. Information on the isotopic integrity of the samples can be found in Supplementary Notes 3.

We performed strontium isotope analysis on samples already analysed for carbon and oxygen isotope ratios, allowing for a direct correlation of potential dietary or environmental shifts to changes in ^87^Sr/^86^Sr ratios. We selected 11 enamel sub-samples based on the adequacy of sample-size and the presence of corresponding peaks in carbon and oxygen values. Strontium isotope analysis of sampled enamel aliquots was conducted at the Curt-Engelhorn-Center for Archaeometry gGmbH, Mannheim (Germany). Further processing of 5 mg of pre-treated powdered enamel, including strontium separation in Teflon columns with Eichrome Sr-spec ion exchange resin, was carried out under clean-lab conditions following the analytical procedures described in Knipper et al.^100^; Blank et al.^101^. ^87^Sr/^86^Sr ratios by High-Resolution Multi Collector-ICP-MS (Neptune). The raw data were corrected according to the exponential mass fractionation law to ^88^Sr/^86^Sr = 8.375209. ^87^Sr/^86^Sr data are reported relative to the NBS 987 standard, which was run along with the samples and yielded ^87^Sr/^86^Sr ratios of 0.71030 ± 0.00001 (n = 7). Blank values during the whole clean lab procedure were less than 50 pg Sr (< 0.1% of the total Sr sample).

Recent studies have shown that enamel formation-related signal dampening can also influence strontium incremental samples, which, upon the application of time-resolute sampling or analytical techniques, have the potential to identify seasonal or even monthly animal mobility patterns^102,103^. However, our analytical strategy prevented the application of inverse modelling to strontium isotopic values.

Our data are correlated to the multi-proxy environmental strontium baseline provided by Frank et al.^47^, which is specifically developed for the Peloponnese. The authors report that bioavailable strontium values from soil leachates and plants correspond well with the values of the underlying geological outcrops, whereas the effects of exogenous mixing inputs, such as precipitation, Saharan dust particles, and sea-spray, play a minimal role in the strontium isotopic composition of the region. The Pindos zone, which can be found predominantly in the west and northwest parts of the Megalopolis Basin (Supplementary Notes 1), is characterized by lower average ^87^Sr/^86^Sr values (between 0.70805 and 0.70855), while higher strontium values are reported for the Tripolis and Arna units, encountered mostly in the north, northeast and east parts of the basin (~ 0.7095)^47^ (Supplementary Notes 1). A single entry from the Megalopolis Basin, collected from Pleistocene clastic sediments of the Pindos geotectonic unit, reports average values of 0.70847 and 0.70835 for plants and soil leachates respectively, (Frank et al.^47^, Supplementary). As a control analysis, we also measured ^87^Sr/^86^Sr ratios in three *Hippopotamus antiquus* individuals from fossiliferous localities within the Megalopolis Basin (Roditi et al. in prep.), to complement the published baseline and define a range of local values for the area. The extant common hippopotamus (*Hippopotamus amphibius*) has small home ranges, estimated to between 3 and 23 km^2^ for both resident and migrant^104^. Assuming similar ecological characteristics for its extinct relative (*Hippopotamus antiquus*), the samples from the hippopotami are likely to track the strontium signature of the local geology. The first specimen originates from the same site (Marathousa 1, Area B; UB2); the second comes from the site of Marathousa 2 (MAR-2)^14^, situated 1.5 km east of MAR-1; the third sampled specimen, an upper third molar, was discovered at Kyparissia 4 (KYP-4)^48,85^, located at the NW part of the basin on top of a Late Cretaceous limestone outcrop of the Pindos geotectonic unit (Fig. 1). The strontium values for the hippopotamus (MAR-1B-8) from MAR-1 corresponded closely to the average ^87^Sr/^86^Sr ratios of plants and soil leachates sampled over the limestone of the Tripolis formation outcrops (Frank et al.^45^, Supplementary), which can be found to the east, north-east, and south of the Megalopolis Basin (Supplementary Notes 1 and Fig. 1). More radiogenic values were observed for the hippopotamus from MAR-2 (MAR-2B-2), corresponding to the Tripolis geotectonic unit. The ^87^Sr/^86^Sr ratio of the latter specimen could have been further influenced by the surrounding lithology, specifically the more radiogenic schist outcrops found east of the Megalopolis basin^47,105^ (Supplementary Fig. 1). Metamorphic clasts (schist, quartzite etc.) mixed with lacustrine sediments have also been observed in the eastern part of the basin. Finally, the ^87^Sr/^86^Sr ratio of the Kyparissia 4 individual (KYP4A-1004) was lower and consistent with the strontium range of the Pindos geotectonic unit.

## Principles of stable isotope analysis

### Carbon isotopes (***δ***^13^C)

The source of carbon atoms in mammals is their dietary intake. In this regard, the ratios of stable carbon isotopes in the tissues of primary consumers reflect the isotopic composition of the ingested vegetation^106^. In terrestrial ecosystems, the isotopic signature of plant carbon varies between plants that utilize the two main photosynthetic pathways, i.e., C_4_ and C_3_^107^. Plant communities following the C_4_ photosynthetic pathway, consist of warm growth season grasses and demonstrate *δ*^13^C values that range in modern environments between − 17‰ and − 9‰ (average –13‰). On the other hand, modern C_3_ plants (trees, shrubs, and cool growth season grasses and sedges) yield *δ*^13^C values ranging from –36‰ to –22‰ (average –27‰)^108,109^. Within plant communities utilizing the C_3_ photosynthetic pathway, the carbon isotopic composition of vegetation is subjected to additional environmentally controlled fractionation, which is influenced by multiple factors, such as the degree of canopy closure, water availability, temperature, light intensity, or atmospheric CO_2_ diffusion^110–112^. The interplay of these factors enables further habitat distinctions within C_3_-dominated ecosystems, wherein negative carbon isotope values (modern *δ*^13^C lower than –30‰) are characteristic of a closed canopy forest and higher values (modern *δ*^13^C between –25‰ and –22‰) characterize C_3_ vegetation under conditions with high irradiance, increased aridity and/or water scarcity^59,112,113^.

The carbon isotopic signature of bioapatite in large herbivores will record the *δ*^13^C of plants with an enrichment in ^13^C of ~ 14.1 ± 0.5‰ due to biomineralization and metabolic processes^114,115^. Additionally, the effects of fossil fuel burning have resulted in the isotopic depletion of modern atmospheric CO_2_ values (–8‰) relative to the Pleistocene (–6.5‰)^116,117^, necessitating the application of a correction factor (+ 1.5‰) in fossil herbivore and plant *δ*^13^C to account for this difference. Upon consideration of the atmospheric CO_2_ correction and the fractionation of diet-enamel bioapatite, the habitats of primary consumers can be categorized into closed canopy forests (< –14.5‰), woodlands-mesic C_3_ grasslands (–14.5‰ to –9.5‰), open woodlands-xeric C_3_ grasslands (–9.5‰ to –6.5‰), mixed C_3_-C_4_ grasslands (–6.5‰ to –1.5‰), and pure C4 grassland (> –1.5‰)^118,119^. Overall, herbivore carbon isotope values become more negative with increasing canopy closure.

### Oxygen isotopes (***δ***^18^Ο)

Oxygen stable isotopic ratios in the skeletal tissues of medium and large mammals are in equilibrium with the isotopic composition of body-water. In turn, the *δ*^18^Ο of body water is influenced by species physiology and drinking behaviour. The main fluxes of oxygen compounds into mammals occur either through diet-water (e.g., leaf water) or by drinking from open water source. Water-dependent taxa (obligate drinkers), such as elephants, track primarily the isotopic composition of the local meteoric water^120^. Variation in the isotopic composition of the latter is governed by several geospatial, climatic, and environmental parameters, for instance the degree of continentality, altitudinal differences of water sources, the amount of local precipitation, surface temperature, as well as differences in the hydrological processes of water bodies (for a detailed overview see Pederzani and Britton^60^). In mid- and high-latitudes, the dominant controls of the *δ*^18^O of meteoric water are surface temperature and local precipitation^121^. In this context, higher *δ*^18^O values are observed in herbivores inhabiting warmer and drier environments and lower values characterize the isotopic composition of animals occupying colder or more humid habitats^60,122^.

Seasonal variability in temperature and rainfall, as well as animal migration patterns, can further influence the *δ*^18^O of mammalian organisms. Sub-annual climate-related fluctuations are expressed in the *δ*^18^O sequential series as a sinusoidally-shaped profile, wherein peaks record the higher temperatures and aridity of summer months, whereas troughs represent the low temperatures and increased rainfall amount in winter^60^. Regarding the influence of animal mobility, non-migrant individuals are expected to display higher intra-tooth *δ*^18^Ο because the isotopic composition of body water tracks primarily the seasonal effects of temperature and precipitation in the utilized water source. In contrast, homogenous oxygen intra-individual profiles are expected in seasonally migrant large herbivores that consume water from different sources along their migration route^45–48^.

### Strontium isotopes (^87^Sr/^86^Sr)

Strontium assimilates into the body of mammals primarily from ingested food sources and to a lesser extent from drinking sources and consumed atmospheric or soil particles. Weathering processes of bedrock materials result in the release of strontium in soils, groundwater, or surface water sources, which is, subsequently, absorbed by plants. Within the geosphere, the strontium isotopic composition of the parent material is a factor of bedrock composition and age. Following the principle of radioactive decay, ^87^Rb (Rubidium) decays to ^87^Sr, with a half-life of 4.96 × 1010^123^. Hence, in general, younger geological units are expected to have lower ^87^Sr/^86^Sr compared to older ones. Within biological tissues, strontium substitutes calcium (Ca) and, in contrast to carbon and oxygen isotopes, strontium isotope ratios are not influenced significantly by physical or chemical alterations between the source and the target tissue (i.e., fractionation)^124^. Thus, the primary factor determining strontium composition within the food chain is the local geological substrate. Strontium in the mineral components of enamel will reflect the average dietary strontium intake during the time of enamel formation^125^.

## Published comparative data

Comparative data for the stable isotope analysis for *P. antiquus* were taken from published works for the following Middle Pleistocene localities of Europe: Mauer (MIS 15, Germany)^58^, Steinheim an der Murr (MIS 11, Germany)^58^, La Polledrara di Cecanibbio (MIS 9, Italy)^53^, Casal de’ Pazzi (MIS 7, Italy)^53,56^, Poggetti Vecchi (MIS 7, Italy)^57^, and Neumark-Nord 1 (MIS 5, Germany)^54^. All of the localities document hominin presence, while La Polledrara, Casal de’ Pazzi, Poggetti Vecchi, and Neumark-Nord 1 additionally preserve direct evidence of *Palaeoloxodon* exploitation in the form of cut marks, human-made bone fractures and/or proboscidean bone artefacts (Konidaris and Tourloukis^42^ and references cited therein; and Gaudzinski-Windheuser et al.^40^ for Neumark-Nord 1).

### Supplementary Information

Supplementary Information.

## Data Availability

The isotope dataset generated during this study, as well as additional associated information and figures, are provided in this published article and the Supplementary Information files.
