# Supplementary material for: Life-history of Palaeoloxodon antiquus reveals Middle Pleistocene glacial refugium in the Megalopolis basin, Greece
Source: Sci Rep. 2024 Jan 16;14:1390. doi: 10.1038/s41598-024-51592-9 (PMC10791645; doi:10.1038/s41598-024-51592-9)
Supplement: Supplementary file 1 — Supplementary Information. [file 41598_2024_51592_MOESM1_ESM.pdf]

1.Supplementary Notes: Geography, geology and climate of the Megalopolis Basin

The Megalopolis Basin is an intramontane, post-orogenic graben located in the central part of the Peloponnese, southern Greece. The area has an altitude of 330–450 meters above sea level (m.a.s.l) and covers a total of 250 km^2, with its longer axis extending approximately 20 km in a NW–SE direction. The Mainalo, Lykaion, and Taygetos mountain ranges border the basin on the east, west, and south respectively. As regards its geology, the basin formed during the Late Miocene–Pliocene on the Mesozoic–Paleogene basement of the Peloponnese, while during the Pleistocene it periodically hosted a large lake[1,2]. In the north-eastern part of the basin, the Plattenkalk series (Mani geotectonic unit) crops out, consisting of Permian to Eocene/Lower Oligocene crystalline carbonates, marbles, and flysch[3]. Outcrops of Carboniferous–Lower Triassic metamorphic rocks, phyllites, schists, and quartzites comprise the Phyllite/Quartzite series (Arna geotectonic unit) and can be found on the south-east and southern extents of the basin[3]. The Tripolis geotectonic zone is present in the eastern, north-eastern, and northern catchment of the graben and consists of the volcano-sedimentary Tyros beds at its base, followed by Upper Triassic–Upper Eocene limestones and dolomites and Upper Eocene–Upper Oligocene flysch[3,4]. Upper Cretaceous to Palaeocene flysch of the Pindos geotectonic zone encompasses the basin in the west and north-west, whereas Upper Cretaceous limestones of the Pindos zone can be found in the east, south-east, west, and north-west (Supplementary Figure 1). The Plio-Pleistocene sedimentary sequence consists of lacustrine, fluvio-lacustrine, and alluvial fan

deposits[1,2,5]. Within this sequence, silts, clays, and sands which intercalate cyclically with lignite seams characterize the Pleistocene lacustrine Marathousa Member (Choremi Formation)[2,5]. Marathousa 1 is stratigraphically placed in this member.

At present, the Megalopolis region is characterized by a Mediterranean climate with humid, cool winters and hot, dry summers. Higher temperatures and lower precipitation are recorded between May and September, whereas the lowest temperatures are observed in January and the highest amount of rainfall occurs in December.

2. Supplementary Notes: Marathousa 1

Marathousa 1 (MAR-1) is a Middle Pleistocene open-air site located at the central-western part of the Megalopolis Basin. The site was discovered in 2013, when stratified faunal remains and lithic artifacts were identified at an exposed section in the Marathousa mine, during a targeted field survey conducted by a joint team of the Ephorate of Palaeoanthropology-Speleology of the Hellenic Ministry of Culture, and the University of Tübingen[6-9]. Subsequent systematic excavations between 2013 and 2019 in two areas —Area A and Area B, located approximately 60 meters apart— revealed a sequence containing micro- and macro-faunal remains[10-13], micro- and macro-floral remains[14], as well as lithic and bone artifacts[15]. The main find-bearing layers (stratigraphic units UA3c/4 and UB4c/5) are situated between Lignite seams IIb and IIIa at an elevation of 350 m.a.s.l. and consist mainly of organic and interclast-rich silty sands[5]. They belong to the same depositional event as evidenced by lithostratigraphic and geochemical data[5], spatial taphonomy[16], as well as archaeological and

palaeontological evidence [11,15]. Electron Spin Resonance (ESR)[17], post-infrared Infrared Stimulated Luminescence[18], magnetostratigraphy[19], and mammal biochronology[13] date the archaeological sequence to ca. 500–400 ka and correlate it to the glacial Marine Isotope Stage (MIS) 12.

The partial skeleton of the straight-tusked elephant was discovered in Area A, at the contact between units UA3c and UA4. Several skeletal elements (vertebrae, ribs, humerus, ulna, femur, tibia, pelvis, carpals, tarsals, metapodials, phalanges) have been excavated in approximate anatomical association, including the cranium bearing both upper third molars[11]. The skeleton belonged to a male individual at its late adulthood, with an upper ontogenetic age-limit between 64–71 years, live skeletal height around 3.7 m at the shoulder, and body mass around 9.0 tonnes[11]. Traces of anthropogenic modifications in the form of cut marks were identified on the astragalus and the tibia, providing direct evidence for the exploitation of the carcass by hominins[11]. This is further confirmed by the spatial and stratigraphic association of the skeleton with lithic artifacts, some of which preserve use-wear traces indicative of butchering activities[16,20]. Further evidence of elephant butchering (e.g., cut marks, breakages in the form of peeling) is present also on bones belonging to another elephant individual from Area B[11], while a thick diaphyseal fragment that may also belong to an elephant is interpreted as a percussor[15].

3. Supplementary Notes: Isotopic signal preservation & integrity

A diaphyseal bone fragment of the MAR-1 (Area A) elephant was initially subjected

to elemental composition analysis (%N and %C yields) and indicated poor collagen preservation; therefore, the present study focused on carbon, oxygen, and strontium isotope analyses on tooth enamel, which, unlike bone and dentine, is considered diagenetically resistant on account of its physical properties, namely its low organic content (<1%) and high crystallinity and, thus, more likely to preserve unaltered the primary isotopic signal[21-24]. Carbon and oxygen stable isotope values for 28 enamel sequential samples from the M^3 of the *P. antiquus* individual from MAR-1 are listed in Supplementary Table 1. To evaluate the fidelity of the isotopic results, we assessed the carbonate content (%$CaCO_3$) of dentine, cementum, and enamel from the same molar[21,22,25,26]. The range of %CO_3 (from 4.32 to 5.20 wt%) in the enamel structural carbonates of the straight-tusked elephant falls within the span of $CaCO_3$ contents of modern enamel (~3.0–5.5 wt%)[27] and close to the range observed in ungulate enamel bioapatite (4.5–5.1 wt%)[28]. Unlike the case of enamel carbonates, the %CO_3 of dentine and cementum are significantly higher (6.99 wt% and 8.74 wt%, respectively), demonstrating that the biogenic signal of these two tissues has been diagenetically altered. Additionally, the presence of intra-tooth, periodic variation in δ-values — likely the result of environmentally induced changes— suggests that the primary isotopic signal is, at least partially, preserved. Such patterns are expected to become homogenized if isotopic re-equilibration has occurred[29,30]. Based on this evidence, we consider the effects of diagenesis in the stable isotope record of the MAR-1 elephant to be negligible and did not obscure the biogenic patterns.

4. Supplementary Discussion: Detailed inter-site comparison of *P. antiquus* isotopic data

In Italy, a C_3-dominated foraging habitat composed of woodland and mesic grassland has been inferred for a *P. antiquus* individual found at the Middle Pleistocene site of Poggetti Vecchi (MIS 7), which yielded a mean $\delta^{13}C$ value, close to the one obtained from the MAR-1 specimen (–11.05 ‰ VPDB)[31]. Carbon isotope analysis demonstrated a wide range of $\delta^{13}C$ values (from –14.7‰ to –9.4‰ VPDB) for the straight-tusked elephant population of Casal de' Pazzi during MIS 7[32,33], whereas sampled specimens from La Polledrara di Cecanibbio (Italy, MIS 9) yielded higher and more constrained $\delta^{13}C$ values (–9‰ to –10.9‰ VPDB)[32]. These data suggest that the latter population was feeding in more open C_3 habitats with water-stress conditions in comparison to the elephant from MAR-1, as well as to the majority of the specimens from Italy correlated with MIS 7[32,33]. At Neumark-Nord 1, $\delta^{13}C$ values for the site's straight-tusked elephant population are depleted when compared to the individual from MAR-1, indicating significant canopy cover during MIS 5[34]. Similarly, analysed specimens from Steinheim an der Murr (MIS 11) and Mauer (MIS 15)[35], yielded lower carbon isotope values compared to those of the MAR-1 specimen, suggesting foraging under increased canopy closure and/or humid environments for the German localities.

The inter-site comparison of oxygen isotopic values shows that the elephant from MAR-1 displays lower average $\delta^{18}O$ compared to most of the Italian *P. antiquus* specimens correlated to MIS 7 and MIS 9, whose values range between +22.8 ‰ and +27.6 ‰ (VSMOW). Higher oxygen isotopic values (+25.5 – +28.0‰) were also obtained for the specimens of Neumark-Nord 1 in Germany during MIS 5. Contrariwise,

the MAR-1 elephant demonstrates a higher $\delta^{18}O$ value compared to the range obtained for the elephants of Steinheim an der Murr and Mauer in Germany. Overall, the comparison of the oxygen isotope data suggests that the interglacial specimens from La Polledrara and Poggetti Vecchi in Italy, as well as the majority of the individuals from Casal de' Pazzi likely experienced warmer or more arid climatic conditions compared to the elephant from MAR-1, whereas cooler or more humid conditions characterize the environment of the interglacial populations from Steinheim an der Murr and Mauer in Germany. The unusually higher oxygen isotopic values of the Neumark Nord-1 population in comparison to both MAR-1 and the other German localities are likely the result of a drier and warmer climate during the Eemian, combined with the effects of evaporative enrichment in the large lake from which the individuals drank water[34,36]. We should note that the effects of continentality could influence the interpretation of the inter-site $\delta^{18}O$ record between Germany and the peri-Mediterranean sites; however, comparisons between Italian and Greek sites should not be significantly subjected to this control.

References

1 Vinken, R. Stratigraphie und Tektonik des Beckens von Megalopolis (Peloponnes, Griechenland). *Geol. Jahrb* **83**, 97–148 (1965).

2 Van Vugt, N., De Bruijn, H., Van Kolfschoten, T., Langereis, C. & Okuda, M. Magneto- and cyclostratigraphy and mammal-fauna's of the Pleistocene lacustrine Megalopolis Basin, Peloponnesos, Greece. *Geologica Ultrajectina* **189**, 69-92 (2000).

3 Tsiftsis, E. V. *Geology and Hydrogeology of the Megalopolis Basin, Peloponnese, Greece*, University of Bristol, (1987).

4 Papanikolaou, D. & Vassilakis, E. Thrust faults and extensional detachment faults in Cretan tectono-stratigraphy: Implications for Middle Miocene extension. *Tectonophysics* **488**, 233–247 (2010).

5 Karkanas, P. *et al.* Sedimentology and micromorphology of the Lower Palaeolithic lakeshore site Marathousa 1, Megalopolis basin, Greece. *Quat. Int.* **497**, 123–136 (2018).
6 Panagopoulou, E. *et al.* Marathousa 1: a new Middle Pleistocene archaeological site from Greece. *Antiquity* **343**, 1–8 (2015).
7 Panagopoulou, E. *et al.* The Lower Palaeolithic site of Marathousa 1, Megalopolis, Greece: overview of the evidence. *Quat. Int.* **497**, 33–46 (2018).
8 Harvati, K., Konidaris, G. & Tourloukis, V. Paleoanthropology at the Gates of Europe: Recent research in Greece in the frame of the PaGE ERC Starting Grant project. *Quat. Int.* **497**, 1–3, doi:10.1016/j.quaint.2018.11.024 (2018).
9 Thompson, N., Tourloukis, V., Panagopoulou, E. & Harvati, K. In search of Pleistocene remains at the gates of Europe: Directed surface survey of the Megalopolis Basin (Greece). *Quat. Int.* **497**, 22–32 (2018).
10 Konidaris, G. E., Athanassiou, A., Panagopoulou, E. & Harvati, K. First record of *Macaca* (Cercopithecidae, Primates) in the Middle Pleistocene of Greece. *J. Hum. Evol.* **162**, 103104, doi:https://doi.org/10.1016/j.jhevol.2021.103104 (2022).
11 Konidaris, G. E. *et al.* The skeleton of a straight-tusked elephant (*Palaeoloxodon antiquus*) and other large mammals from the Middle Pleistocene butchering locality Marathousa 1 (Megalopolis Basin, Greece): preliminary results. *Quat. Int.* **497**, 65–84 (2018).
12 Michailidis, D., Konidaris, G. E., Athanassiou, A., Panagopoulou, E. & Harvati, K. The ornithological remains from Marathousa 1 (Middle Pleistocene; Megalopolis basin, Greece). *Quat. Int.* **497**, 85–94 (2018).
13 Doukas, C., van Kolfschoten, T., Papayianni, K., Panagopoulou, E. & Harvati, K. The small mammal fauna from the Palaeolithic site Marathousa 1 (Greece). *Quat. Int.* **497**, 95–107 (2018).
14 Field, M. H. *et al.* A palaeoenvironmental reconstruction (based on palaeobotanical data and diatoms) of the Middle Pleistocene elephant (*Palaeoloxodon antiquus*) butchery site at Marathousa, Megalopolis, Greece. *Quat. Int.* **497**, 108–122 (2018).
15 Tourloukis, V. *et al.* Lithic artifacts and bone tools from the Lower Palaeolithic site Marathousa 1, Megalopolis, Greece: Preliminary results. *Quat. Int.* **497**, 47–64, doi:https://doi.org/10.1016/j.quaint.2018.05.043 (2018).
16 Giusti, D. *et al.* Beyond maps: patterns of formation processes at the Middle Pleistocene open-air site of Marathousa 1, Megalopolis Basin, Greece. *Quat. Int.* **497**, 137–153 (2018).
17 Blackwell, B. A. *et al.* ESR dating ungulate teeth and molluscs from the Paleolithic site Marathousa 1, Megalopolis Basin, Greece. *Quaternary* **1**, 22 (2018).
18 Jacobs, Z. *et al.* Optical dating of K-feldspar grains from Middle Pleistocene lacustrine sediment at Marathousa 1 (Greece). *Quat. Int.* **497**, 170–177, doi:https://doi.org/10.1016/j.quaint.2018.06.029 (2018).
19 Tourloukis, V. *et al.* Magnetostratigraphic and chronostratigraphic constraints on the Marathousa 1 Lower Palaeolithic site and the Middle Pleistocene deposits of the Megalopolis basin, Greece. *Quat. Int.* **497**, 154–169, doi:https://doi.org/10.1016/j.quaint.2018.03.043 (2018).
20 Guibert-Cardin, J. *et al.* The function of small tools in Europe during the Middle Pleistocene: The case of Marathousa 1 (Megalopolis, Greece). *Journal of lithic studies* **9** (2022).

21 Lee-Thorp, J. A. & Van der Merwe, N. J. Aspects of the chemistry of modern and fossil biological apatites. *J. Archaeol. Sci.* **18**, 343–354 (1991).
22 van der Merwe, N. J. & Medina, E. The canopy effect, carbon isotope ratios and foodwebs in Amazonia. *J. Archaeol. Sci.* **18**, 249–259 (1991).
23 Zazzo, A., Lécuyer, C. & Mariotti, A. Experimentally-controlled carbon and oxygen isotope exchange between bioapatites and water under inorganic and microbially-mediated conditions. *Geochim. Cosmochim. Acta* **68**, 1–12, doi:https://doi.org/10.1016/S0016-7037(03)00278-3 (2004).
24 Bocherens, H., Sandrock, O., Kullmer, O. & Schrenk, F. Hominin palaeoecology in Late Pliocene Malawi: First insights from isotopes (^{13}C, ^{18}O) in mammal teeth. *S. Afr. J. Sci.* **107**, 1-6 (2011).
25 Ayliffe, L., Chivas, A. R. & Leakey, M. The retention of primary oxygen isotope compositions of fossil elephant skeletal phosphate. *Geochim. Cosmochim. Acta* **58**, 5291–5298 (1994).
26 Wang, Y. & Cerling, T. E. A model of fossil tooth and bone diagenesis: implications for paleodiet reconstruction from stable isotopes. *Palaeogeogr. Palaeoclimatol. Palaeoecol.* **107**, 281–289 (1994).
27 Sydney-Zax, M., Mayer, I. & Deutsch, D. Carbonate content in developing human and bovine enamel. *J. Dent. Res.* **70**, 913–916, doi:10.1177/00220345910700051001 (1991).
28 Rink, W. J. & Schwarcz, H. P. Tests for diagenesis in tooth enamel: ESR dating signals and carbonate contents. *J. Archaeol. Sci.* **22**, 251–255, doi:https://doi.org/10.1006/jasc.1995.0026 (1995).
29 Goedert, J. *et al.* Preliminary investigation of seasonal patterns recorded in the oxygen isotope compositions of theropod dinosaur tooth enamel *Palaios* **31**, 10–19 (2016).
30 Owocki, K., Kremer, B., Cotte, M. & Bocherens, H. Diet preferences and climate inferred from oxygen and carbon isotopes of tooth enamel of *Tarbosaurus bataar* (Nemegt Formation, Upper Cretaceous, Mongolia). *Palaeogeogr. Palaeoclimatol. Palaeoecol.* **537**, 109190, doi:https://doi.org/10.1016/j.palaeo.2019.05.012 (2020).
31 Capalbo, C. Multiproxy-Based reconstruction of the feeding habits from the late Middle Pleistocene straight-tusked elephant population of Poggetti Vecchi (Southern Tuscany, Italy). *Alp. Mediterr. Quat.* **31**, 113–119 (2018).
32 Palombo, M. R. *et al.* Coupling tooth microwear and stable isotope analyses for palaeodiet reconstruction: the case study of Late Middle Pleistocene *Elephas (Palaeoloxodon) antiquus* teeth from Central Italy (Rome area). *Quat. Int.* **126**, 153–170 (2005).
33 Briatico, G. & Bocherens, H. Middle Pleistocene ecology in central Italy. New isotopic insights from fauna tooth enamel of Casal de'Pazzi (Rome, Italy). *J. Mediterr. Earth Sci.* **15** (2023).
34 Grube, R., Palombo, M., Iacumin, P. & Di Matteo, A. What did the fossil elephants from Neumark-Nord eat in *Elefantenreich. Eine Fossilwelt in Europa* (ed. H. Meller) 253–272 (Landesamt für Denkmalpflege und Archäologie Sachsen-Anhalt, 2010).
35 Pushkina, D., Bocherens, H. & Ziegler, R. Unexpected palaeoecological features of the Middle and Late Pleistocene large herbivores in southwestern Germany revealed by stable isotopic abundances in tooth enamel. *Quat. Int.* **339-340**, 164–178, doi:https://doi.org/10.1016/j.quaint.2013.12.033 (2014).

36 Pop, E. & Bakels, C. Semi-open environmental conditions during phases of hominin occupation at the Eemian Interglacial basin site Neumark-Nord 2 and its wider environment. *Quaternary Science Reviews* **117**, 72-81, doi:https://doi.org/10.1016/j.quascirev.2015.03.020 (2015).

Supplementary Figures & Tables

Supplementary Figure 1: Simplified geological map of south-western Peloponnese showing the location of Marathousa 1. The figure was generated using Adobe Illustrator CC 2017 (https://www.adobe.com/).

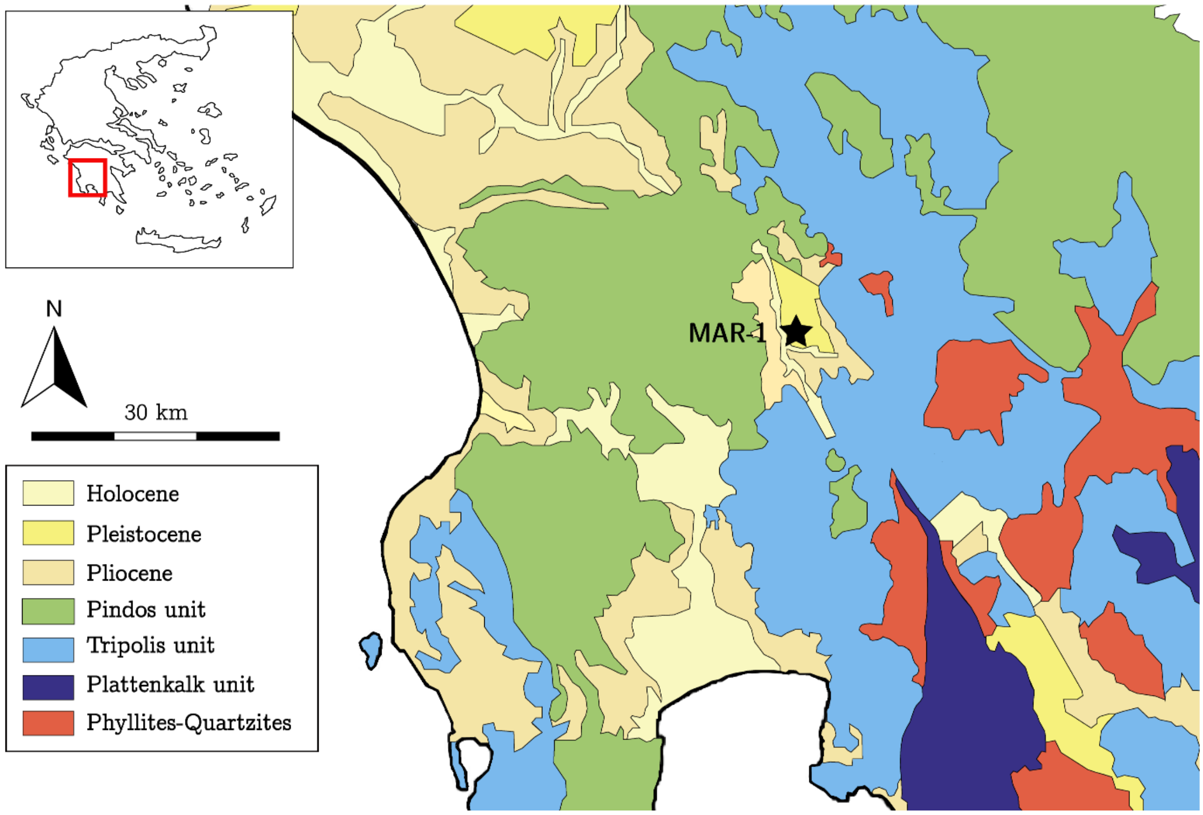

Supplementary Table 1. Main results of the incremental C, O, and Sr isotopic measurements from the right upper third molar of *Palaeoloxodon antiquus,* and Sr results from the teeth of *Hippopotamus antiquus.*

Taxon	Specimen	Sample	$\delta^{13}C$ (VPDB)	$\delta^{18}O$ (VSMOW)	$^{87}Sr/^{86}Sr$	CO_3 (%$CaCO_3$)	Distance from cervix (mm)
Palaeoloxodon antiquus	MAR-1A-5	MAR-1-942-676.1	-11.1	24.2	0.7087	4.3	87
		MAR-1-942-676.2	-11.5	23.9		4.5	84
		MAR-1-942-676.3	-12.1	23.5	0.7088	4.4	82
		MAR-1-942-676.4	-11.5	23.8		4.8	78
		MAR-1-942-676.5	-12.0	23.9	0.7088	4.5	76
		MAR-1-942-676.6	-11.9	23.6		4.3	73
		MAR-1-942-676.7	-11.5	23.2	0.7088	4.4	70
		MAR-1-942-676.8	-11.5	23.4		4.5	67
		MAR-1-942-676.9	-11.3	23.8	0.7088	4.6	64
		MAR-1-942-676.10	-11.4	23.3		4.5	61
		MAR-1-942-676.11	-11.3	23.2	0.7089	4.6	58
		MAR-1-942-676.12	-11.1	23.9		4.7	55
		MAR-1-942-676.13	-10.8	23.3		4.9	52
		MAR-1-942-676.14	-10.8	23.7		4.8	49
		MAR-1-942-676.15	-10.8	23.7	0.7088	4.7	46
		MAR-1-942-676.16	-11.1	23.4		4.7	43
		MAR-1-942-676.17	-10.4	23.4		5.2	40
		MAR-1-942-676.18	-10.9	23.4		5.1	37
		MAR-1-942-676.19	-11.4	22.9	0.7089	4.8	34
		MAR-1-942-676.20	-11.3	23.4		4.8	31
		MAR-1-942-676.21	-11.6	23.6	0.7088	4.6	28
		MAR-1-942-676.22	-11.4	23.4		4.5	26
		MAR-1-942-676.23	-11.1	23.6		4.8	23
		MAR-1-942-676.24	-10.8	23.0		5.2	20
		MAR-1-942-676.25	-10.8	23.8		5.2	17
		MAR-1-942-676.26	-11.3	23.0	0.7088	4.9	15
		MAR-1-942-676.27	-11.0	23.6		4.8	12
		MAR-1-942-676.28	-10.5	24.3	0.7084	5.1	9
		MAR-1-942-676.dent	1.9	24.9		7.0	
		MAR-1-942-676.cem	-3.9	24.0		8.7	
Hippopotamus antiquus	MAR-1B-8	-			0.7087		
	MAR-2B-2	-			0.7091		
	KYP4A-1004	-			0.7084		

Supplementary table 2: Published SI data on *Palaeoloxodon antiquus* used for inter-site comparison.

Region	Site	MIS	$\delta^{13}C$ (VPDB)	$\delta^{18}O$ (VSMOW)	Sampling	Reference
Italy	Casal de' Pazzi	7	-11.9	26.1	Bulk sample	Palombo et al. 2005[4]
			-9.9	27.2		
			-11.3	27.0		
			-12.1	25.9		
			-11.4	25.9		
			-11.3	26.3		
			-11.3	26.0		
			-13.0	26.3		
			-13.7	27.2		
			-13.3	27.1		
			-13.7	27.0		
			-13.2	23.9		Briatico & Bocherens 2023[1]
			-13.3	24.6		
			-11.5	23.5		
			-14.6	24.9		
			-14.7	24.7		
			-12.9	23.9		
			-10.9	25.2		
			-12.6	22.8		
			-9.4	24.8		
	La Polledrara di Ceccanibio	9	-9.0	24.8	Bulk sample	Palombo et al. 2005[4]
			-10.2	27.4		
			-10.9	26.8		
			-10.8	26.5		
			-10.3	26.8		
			-10.6	27.0		
			-10.7	27.6		
			-10.4	27.0		
			-10.1	26.8		
			-9.8	27.0		
			-10.6	26.8		
			-10.6	26.7		
	Poggetti Vecchi	7	-11.1	26.7	Bulk sample	Capalbo et al. 2018[2]
Germany	Neumark Nord-1	5e	-12.2	26.9	Bulk sample	Grube et al. 2010[3]
			-11.2	27.5		
			-13.1	25.5		
			-11.7	27.5		
			-11.7	27.6		
			-12.6	27.0		
			-14.1	27.9		
			-13.4	27.6		
			-12.8	27.9		
			-13.9	28.0		
			-12.4	26.8		
			-12.3	26.6		
			-11.9	26.3		
			-13.3	25.5		
			-13.1	25.8		
	Mauer	15	-13.9	21.8	Bulk sample	Pushkina et al. 2014[5]
			-12.4	22.5		
			-12.4	20.3		
	Steinheim an der Murr	11	-13.2	20.5	Bulk sample	Pushkina et al. 2014[5]
			-13.1	21.7		
			-13.0	21.1		
			-12.9	21.9		
			-12.6	21.4		

References

1. Briatico, G. and Bocherens, H. 2023. Middle Pleistocene ecology in central Italy. New isotopic insights from fauna tooth enamel of Casal de'Pazzi (Rome, Italy). Journal of Mediterranean Earth Sciences, 15.

2. Capalbo, C. 2018. Multiproxy-Based reconstruction of the feeding habits from the late Middle Pleistocene straight-tusked elephant population of Poggetti Vecchi (Southern Tuscany, Italy). Alpine and Mediterranean Quaternary, 31, pp. 113–119.

3. Grube, R., Palombo, M., Iacumin, P. and Di Matteo, A. 2010. What did the fossil elephants from Neumark-Nord eat, in: Meller, H.(ed.) Elefantenreich. Eine Fossilwelt in Europa. Landesamt für Denkmalpflege und Archäologie Sachsen-Anhalt, Halle. Halle: Landesamt für Denkmalpflege und Archäologie Sachsen-Anhalt.

4. Palombo, M. R., Filippi, M. L., Iacumin, P., Longinelli, A., Barbieri, M. and Maras, A. 2005. Coupling tooth microwear and stable isotope analyses for palaeodiet reconstruction: the case study of Late Middle Pleistocene *Elephas (Palaeoloxodon) antiquus* teeth from Central Italy (Rome area). Quaternary International, 126, pp. 153–170.

5. Pushkina, D., Bocherens, H. and Ziegler, R. 2014. Unexpected palaeoecological features of the Middle and Late Pleistocene large herbivores in southwestern Germany revealed by stable isotopic abundances in tooth enamel. Quaternary International, 339-340, pp. 164–178.
